# Synthesis and Study of Sorption Properties of Zinc-Imprinted Polymer

**DOI:** 10.3390/polym16243545

**Published:** 2024-12-19

**Authors:** Alma Khassenovna Zhakina, Yevgeniy Petrovich Vassilets, Oxana Vasilievna Arnt, Almat Maulenuly Zhakin

**Affiliations:** Limited Liability Partnership «Institute of Organic Synthesis and Coal Chemistry of the Republic of Kazakhstan», Karaganda 100008, Kazakhstan; vassilets88@mail.ru (Y.P.V.); oxana230590@mail.ru (O.V.A.); zhakin-almat@mail.ru (A.M.Z.)

**Keywords:** imprinted polymer, humic acids, zinc ions, adsorption, purification, selectivity

## Abstract

Zinc-imprinted polymer (ZnIP) and non-imprinted polymer (NIP) were synthesized by radical polymerization, and their properties were studied. The novelty of the work lies in the use of humic acids isolated from coals of the Shubarkol deposit (Karaganda, Kazakhstan) as a basis for the imprinted polymer matrix, with methacrylic acid and ethylene glycol dimethacrylate as a functional monomer and a cross-linking agent, respectively. The composition and structure of ZnIP and NIP were characterized using various physicochemical methods. The specific surface area of ZnIP determined by the BET method was 40.60 ± 0.4 m^2^/g, which is almost twice as high as the similar indicator for NIP (21.50 ± 0.3 m^2^/g). In sorption tests of solutions with bimetallic ions, ZnIP demonstrates higher adsorption: 96.15% for Zn^2+^ and 74.88% for Pb^2+^, while NIP adsorbs only 81.33% and 60.11%, respectively. Sorption on both polymers is described by a pseudo-first-order equation (r > 0.99). The distribution coefficients for ZnIP are higher than for NIP. ZnIP has a relative selectivity that exceeds NIP by 2.90 times. The research results indicate the promise of using ZnIP for the selective removal of zinc ions from solutions of multicomponent systems, including wastewater, making it a valuable material for solving environmental and technological problems.

## 1. Introduction

The pollution of water resources by ecotoxicants is a serious environmental problem. Metals are particularly dangerous among substances that harmful to human health. If the permissible concentrations are exceeded, they become toxic, which has led many countries to establish strict environmental standards for the concentration of metal ions in water. To reduce their level, various methods are used, such as chemical precipitation, ion exchange, adsorption, membrane processes and electrodialysis.

However, these methods have their limitations, as they are associated with high operating costs, or they may not be effective enough to meet the regulatory requirements for water quality. A successful solution to environmental protection issues today is possible only through advanced technologies and materials, including new sorption materials, since adsorption is recognized as the most effective method for removing pollutants from water.

Modern research shows great potential for rapidly developing molecular imprinting technologies for creating a new generation of sorption materials [1,2,3,4,5,6,7,8,9,10,11,12,13,14,15]. A particularly promising area is the development of imprinted polymers based on biopolymers, which can be used for the selective separation of complex mixtures [16,17,18,19]. The interest in research is due to the fact that imprinted polymers are used not only in scientific research but also in chemical, medical [9,12,14], pharmaceutical [20,21] and biotechnological [22,23,24,25] industries, especially at the stages of purification of end products. A key aspect of using molecularly imprinted polymers in sorption materials is their high efficiency in removing pollutants, including trace concentrations, and their exceptional stability under harsh conditions (high temperature, pressure, organic solvents), which simplifies the process of water purification [3,4,6,7,9,10,12,13,14,26] and makes them valuable for solving environmental problems [27,28,29].

The living conditions we live in are characterized by the ever increasing pollution of water with heavy metals, which poses a serious threat to the environment. Among these metals, zinc (Zn) is of particular interest. Zinc is a metal that easily enters wastewater from industrial processes, which has a negative impact on the environment. Therefore, it is necessary to take measures to purify it from wastewater. In [4,29], Wirawan et al. synthesized a novel Zn(II)-ionic imprinted polymer by precipitation polymerization using 8-hydroxyquinoline (8HQ) as a ligand, methacrylic acid (MAA) as a functional monomer and ethylene glycol dimethacrylate (EGDMA) as a crosslinking agent. The optimal conditions for zinc adsorption on the ion-imprinted polymer were determined. In [30], the author synthesized and characterized a zinc (II)-centered [Zn(maa)_2_(vim)_2_] monomer complex. The author claims that Zn(II)-IIP can be a suitable sorbent for the removal of Zn(II) ions from aqueous solutions.

In the work of Zia [31], a polymer with ionic imprinting of Zn(II) [Zn(II) IIP] was prepared by subsequent precipitation polymerization using acrylonitrile as a functional monomer, N,N′-methylenebis(acrylamide) as a crosslinking agent and potassium persulfate as a thermal initiator. The obtained polymer demonstrated a high tendency for selective recombination with Zn(II) ions.

In recent years, research has been actively conducted, aimed at creating new sorbents based on humic acids [32,33,34,35,36]. Due to their porous structure, humic acids have good sorption properties, which makes them promising for water purification. The use of humic acid-based sorbents makes it possible to remove various pollutants from wastewater, including heavy metals. Special attention is paid to the removal of zinc, which is often found in wastewater and poses a serious environmental hazard. Zinc salts can have a negative impact on ecosystems, and its excess or deficiency can disrupt vital processes in organisms. Therefore, zinc removal from water is an important factor in water treatment technologies, especially in industrial settings.

Previously, we synthesized imprinted polymers based on humic acids isolated from the coals of the Shubarkol deposit (Karaganda, Kazakhstan) to remove heavy metals from water [37,38].

This work is a continuation of research on the synthesis of imprinted polymers based on humic acids and is devoted to the synthesis of zinc-imprinted polymers based on humic acids and functional monomer (methacrylic acid), as well as the study of their sorption properties. The possibility of using a zinc-imprinted polymer as a selective sorption material for water purification from zinc ions is evaluated.

## 2. Materials and Methods

### 2.1. Chemicals

In the synthesis of zinc-imprinted polymer (ZnIP) and non-imprinted polymer (NIP), humic acids (HAs) obtained from oxidized coal from the Shubarkol deposit (Karaganda, Kazakhstan) [38]. Methacrylic acid (MAA; Merck, Darmstadt, Germany; CAS:79-41-4) was used as a functional monomer. Ethylene glycol dimethacrylate (EGDMA; Merck, Darmstadt, Germany; CAS:97-90-5) was used as a crosslinking agent, while benzoyl peroxide (BP; Merck, Darmstadt, Germany; CAS:94-36-0) was used as a reaction initiator. Zinc acetate (Merck, Darmstadt, Germany; CAS:5970-45-6) was used to obtain the prints. The objects of sorption research were aqueous solutions of zinc acetate, which were prepared by dissolving precise Zn(CH_3_COO)_2_·H_2_O attachments in distilled water.

### 2.2. Synthesis of Zinc-Imprinted Polymer

The synthesis of zinc-imprinted polymer (ZnIP) was carried out according to the non-covalent imprinting technique developed by us using radical polymerization. A solution of Zn(CH_3_COO)_2_ (0.1 mmol) was added to an aqueous solution of HA (1 mmol) and treated with ultrasound (US) for 30 min. Further, the mixture was kept under stirring for 6 h until a stable pre-polymerization complex was formed between HA molecules and Zn^2+^ ions. Then a functional monomer (methacrylic acid, 1 mmol), a crosslinking agent (EGDMA, 10 mmol) and an initiator (BP, 0.1 mmol) were introduced into this complex. To prevent oxygen exposure, the reaction mixture was purged with argon for 15 min. Polymerization was carried out for 180 min in a thermostat (Termex, Tomsk, Russia) at a temperature of 60 °C. After polymerization was completed, the resulting product was centrifuged (Hermle centrifuge, Labortechnik GmbH, Wechingen, Germany) at a speed of 14,000 rpm, washed with distilled water to a neutral medium, dried, crushed (IKA shredder, Werke GmbH & Co., Staufen, Germany), sieved using laboratory sieves, and a fraction with a particle size of 200–400 microns was selected. Zn^2+^ ions were removed from the crushed product by acid hydrolysis with 0.1 N HCl solution, heated to 50–60 °C and kept for 30 min. The resulting product was filtered, and the precipitate was washed with distilled water until the Cl^−^ ions disappeared.

A non-imprinted polymer (NIP) was synthesized using a similar technique, but without the addition of Zn^2+^ ions.

### 2.3. Characterization Studies

The structures of ZnIP and NIP were studied using infrared Fourier spectroscopy FSM-1201 (Infraspec Company, St. Petersburg, Russia) in the wave number range of 4000–400 cm^−1^, with the error not exceeding 2 cm^−1^.

To assess the content of oxygen-containing groups in ZnIP and NIP, the conductometric method of reverse titration using the laboratory conductometer Anion-4100 (Infraspak-Analyte, Novosibirsk, Russia) was used. The measurements were carried out sequentially on three hitches, and the average value was obtained from three experiments.

Elemental analysis for the content of carbon, hydrogen, nitrogen and oxygen in ZnIP and NIP was performed on an elemental analyzer (Elementar Unicube, Langenselbold, Germany).

The surface morphology of ZnIP and NIP was characterized using scanning electron microscopy (MIRA 3, Tescan Orsay Holding, Brno-Kohoutovice, Czech Republic), equipped with detectors registering various signals. Topographic contrast images were obtained using secondary electron detectors, and the elemental composition of ZnIP and NIP was determined using X-ray energy dispersive microanalysis.

The Ultrasonic Homogenizer JY92-IIDN, with a maximum power of 900 W and a frequency of 25 kHz (Scientz, Ningbo, China), was used as an ultrasound source. The equipment is equipped with a 7-inch touchscreen to control the instruments. The ultrasonic power step is smoothly adjustable by 1%, with pulse and continuous operation, as well as with a testing function. All operating parameters of the device, including temperature, can be adjusted individually.

The thermal stability of ZnIP and NIP was studied by differential thermal analysis (DTA) using a synchronous thermogravimetric differential analyzer Perkin Elmer STA 6000 (Shelton, CT, USA) in the measurement range: T_melt_. up to 800 °C in a nitrogen atmosphere, υ = 10°/min.

The specific surface area of ZnIP and NIP was measured using the Sorbi-MS measuring complex (META, St. Petersburg, Russia) using the Brunaer–Emmett–Taylor (BET) method, and the pore distribution was determined using the Barrett–Joyner–Halenda method. Thermal testing of the samples was carried out using the SorbiPrep device. Nitrogen of high purity, Grade A (99.99%), was used as an adsorbate. The measurement was carried out at a liquid nitrogen temperature of 77 K.

The equilibrium concentrations of Zn^2+^ ions in the filtrate were determined using an inductively coupled plasma iCAR6500 atomic emission spectrometer (SPECTRO ARCOS EOP SPECTRO Analytical instruments GmbH, Kleve, Germany).

### 2.4. Adsorption of Zn^2+^ Ions

Sorption by ZnIP was carried out in a static mode. To do this, a 0.1 g suspension was placed in a flat-bottomed flask with a lapped stopper. Next, 25 mL of zinc acetate solutions with concentrations increasing from 100 to 2000 mg/L were poured into the flasks. The solutions were mixed for 6 h using a laboratory shaker (PE-6410, St. Petersburg, Russia). After the adsorption equilibrium was established, the product was separated by filtration. The equilibrium concentrations of Zn^2+^ ions in the filtrate were determined using an inductively coupled plasma iCAR6500 atomic emission spectrometer (SPECTRO ARCOS EOP SPECTRO Analytical instruments GmbH, Germany). The number of Zn^2+^ ions adsorbed on ZnIP was calculated as the ratio of the difference in concentrations of Zn^2+^ ions in solution before and after sorption, related to the unit mass of the polymer:

(1) $$A=\frac{{(C}_{0}-C_{P})\cdot V}{m}$$

where A—the number of sorbed Zn^2+^ ions (mmol/g); C_0_—the concentration of Zn^2+^ ions in the initial solution before sorption (mmol/L); C_p_—the equilibrium concentration of Zn^2+^ ions in the solution after sorption (mmol/L); m—the mass of ZnIP (g); V—the volume of the analyzed solution (mL).

According to the obtained values of the sorption capacity (A, mmol/g) adsorption isotherms were constructed in the studied ZnIP at different equilibrium concentrations of Zn^2+^ ions (C_p_, mg/L).

At the same time, the sorption characteristics were calculated: the degree of extraction (R, %), the distribution coefficient (D) and the imprinting factor (IF), according to the following equations:

(2) $$R=\frac{C_{0}-C_{p}}{C_{0}}\cdot 100\%$$

(3) $$D=\frac{R}{\left(100-R\right)}\cdot \frac{V}{m}$$

(4) $$IF=\frac{D_{ZnIP}}{D_{NIP}}$$

Similarly, the sorption properties for NIP were studied using the above method.

The effects of pH and time on the adsorption capacity of ZnIP and NIP were studied. The effect of pH on adsorption capacity was studied in the range of 3.0–9.0. The measurements were repeated 3 times, and the results were analyzed. To construct kinetic adsorption curves, zinc acetate solutions were prepared. Then, 0.1 g of ZnIP (NIP) was added and mixed on a laboratory shaker (PE-6410, St. Petersburg, Russia). Samples were taken at 5, 10, 20, 30, 40, and 60 min. According to the obtained values of the degree of sorption of Zn^2+^ ions, isotherms were constructed for the studied ZnIP and NIP at different times.

The obtained sorption isotherms can theoretically be described on the basis of two equations:

(1) The Langmuir monomolecular sorption model equation describing the sorption isotherm over the entire concentration range:

(5) $$A=\frac{A_{max}\cdot (K_{sorb}\cdot C_{p})}{1+K_{sorb}\cdot C_{p}}$$

or in a linear form:

(6) $$\frac{C_{p}}{A}=\frac{1}{K_{sorb}\cdot A_{max}}+\frac{1}{A_{max}}\cdot C_{p}$$

where K_sorb_—sorption constant, l/mmol; A—maximum sorption capacity, mmol/g.

(2) The empirical Freundlich equation, which is usually used to represent the average section of the sorption isotherm:

(7) $$A=K_{Fr}\cdot C_{p}^{1/n}$$

or in a linear form:

(8) $$lgA=lgK_{Fr}+\frac{1}{n}\cdot lgC_{p}$$

where K_Fr_—Freundlich’s constant, which represents the amount of an adsorption substance at an equilibrium concentration equal to one; 1⁄n—a constant whose value is equal to the correct fraction.

To determine the pseudo-order, a graphical method was chosen using kinetic equations for reactions of integer order—the first (9), second (10) and third (11):

(9) $$lnC=lnC_{0}-k_{1}t$$

(10) $$\frac{1}{C}=k_{2}t+\frac{1}{C_{0}^{2}}$$

(11) $$\frac{1}{C^{2}}=k_{3}t+\frac{1}{C_{0}^{2}}$$

where k_i_—the rate constant of the ith order of the reaction, and t is the reaction time (min).

### 2.5. Selectivity Studies

The selectivity of ZnIP and NIP was investigated by conducting competitive adsorption experiments. For this purpose, solutions containing ions of two metals (Zn^2+^ and Pb^2+^) were used, where Pb^2+^ ions acted as a competitor to evaluate the abilities of ZnIP and NIP for selective extraction. To determine selectivity, 25 mL of zinc and lead acetate solution with a concentration of 0.001 mol-eq/L was added to 0.1 g of synthesized ZnIP and NIP. Sorption was carried out in static mode for 12 h, at a temperature of 22 ± 2 °C and pH 5, which is optimal for this process. After reaching adsorption equilibrium, the content of non-adsorbed Zn^2+^ and Pb^2+^ ions in solution was determined using an atomic emission spectrometer with inductively coupled plasma ICAR6500 DUOLA. Based on the data obtained, the distribution coefficient (K_d_), the selectivity factor k of Zn^2+^ ions with respect to Pb^2+^ ions and relative selectivity were calculated.

## 3. Results and Discussion

### 3.1. Characterization Analysis

The zinc-imprinted polymer (ZnIP) was prepared in three stages. The first stage is the synthesis of a pre-polymerization complex consisting of a polymer (humic acids, HAs) and Zn^2+^ ions under the action of ultrasound (US). In the second stage, a functional monomer (methacrylic acid, MAA), a crosslinking agent (ethylene glycol dimethacrylate, EGDMA) and an initiator of the polymerization reaction (benzoyl peroxide, BP) were added to the pre-polymerization complex. The mixture was subjected to heat treatment at 60 °C for 180 min. The third stage is the removal of Zn^2+^ ions from the polymer grid by acid hydrolysis. A non-imprinted polymer (NIP) was synthesized under similar conditions without the participation of Zn^2+^ ions.

**Figure 1:**
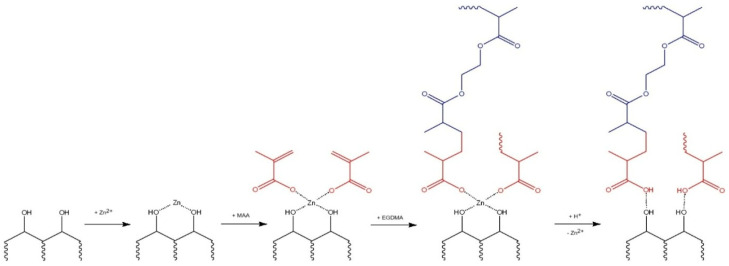
ZnIP synthesis scheme.

**Figure 2:**
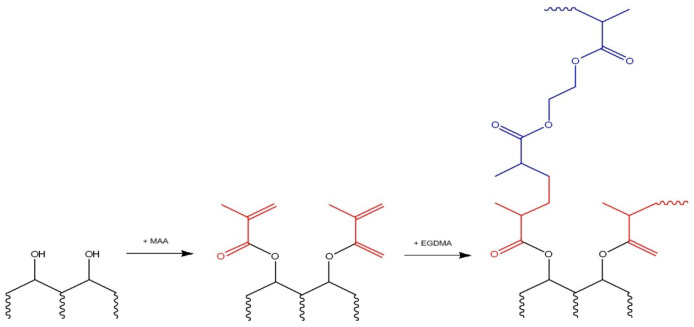
NIP synthesis scheme.

Figure 1 and Figure 2 show the synthesis schemes of ZnIP and NIP.

Table 1 shows the initial components used for the synthesis of ZnIP and NIP.

The results of chemical studies of synthesized ZnIP and NIP are confirmed by the data from elemental analysis, IR spectroscopy, conductometry, electron microscopy, thermal analysis and atomic emission spectroscopy. The physico-chemical characteristics of ZnIP and NIP are given in Table 2.

The results of the elemental analysis (Table 2) showed a decrease in the oxygen content of ZnIP by 4.12% compared to NIP. This indicates the interaction of Zn^2+^ ions with carboxyl and hydroxyl groups of HA, which leads to a decrease in the number of these groups in the ZnIP structure. The decrease in oxygen-containing groups in ZnIP also confirms the possibility of Zn^2+^ ion binding through the mechanism of complexation. The total content of oxygen-containing groups (Σ(COOH+OH)) in ZnIP was 4.43 mg-eq/g, whereas in NIP it was 4.95 mg-eq/g. The yields of the synthesized polymers were similar: for ZnIP, the yield was 76.54%, and for NIP, it was 77.98%. This indicates that the imprinting process does not affect the output of products.

**Figure 3:**
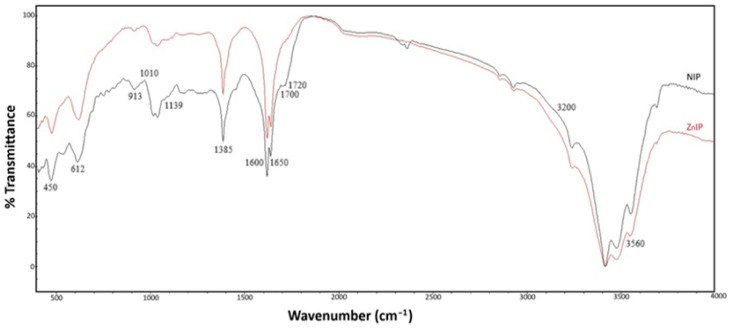
IR spectra of ZnIP and NIP.

The infrared spectra of ZnIP and NIP are shown in Figure 3.

The IR spectra of ZnIP and NIP were very similar, since these samples were synthesized using the same methodology and starting materials. The images of ZnIP and NIP samples showed peaks in the region of 1010–1139 cm^−1^, which correspond to the stretching of the C–O bonds of carbohydrates, alcohol and ester groups, indicating the presence of these functional groups in the ZnIP and NIP polymers. The bands with a maximum at 913 cm^−1^ are due to the presence of substituted aromatic structures. The changes in the region of 1385 cm^−1^ can be explained by the destructive processes that affect the structure of both ZnIP and NIP polymers, leading to a reduction in the length of the aliphatic chain and an increase in the number of ring –CH_3_ groups. The appearance of the band in the region of 1600–1650 cm^−1^ in both ZnIP and NIP is associated with vibrations of the C=C double bond of methacrylic acid and aromatic fragments of HA. In the region of 1700–1720 cm^−1^, an increase in transparency (decrease in absorption) of vibrations of the carboxyl group is observed, likely associated with the formation of zinc salt in the form of O–Zn–O bridges. Their presence in the IR spectrum of NIP, but absence in the spectrum of ZnIP, indicates that a coordination complex is formed in ZnIP, which can complicate the detection of carboxyl groups. Stretching of the peaks in the region of 3200–3560 cm^−1^, characteristic of hydroxyl groups, may indicate possible binding of zinc ions in the ZnIP sample by ion exchange and complexation mechanisms. Clearly defined peaks in the region of 450 and 612 cm^−1^ are the result of superposition of deformation vibrations (σ C–H) in the aromatic rings of both ZnIP and NIP.

**Figure 4:**
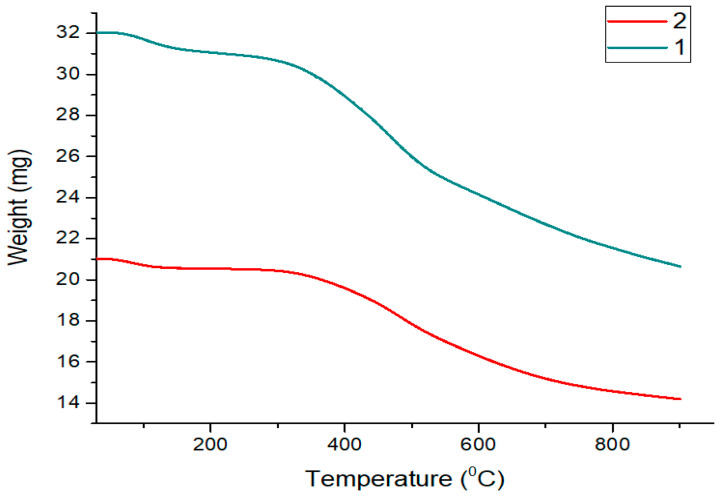
Thermal stability of ZnIP and NIP.

The results of the study on the thermal resistance of ZnIP and NIP are shown in Figure 4.

As can be seen from Figure 4, the thermal stability of ZnIP and NIP is similar, which indicates the similarity of their physico-chemical properties. A slight endothermic effect and weight loss up to 210 °C indicate water desorption. At a temperature of 350–550 °C, a significant weight loss was recorded (32.59 wt.% for ZnIP and 35.57 wt.% for NIP), which is due to the thermal effects of the components, likely compounds with organic fragments of MAA, EGDMA and HA. With a further increase in temperature to 800 °C, no mass loss is observed, which indicates the completion of the active stage of pyrolysis and the appearance of more stable residues consisting of mineral components of HA.

**Figure 5:**
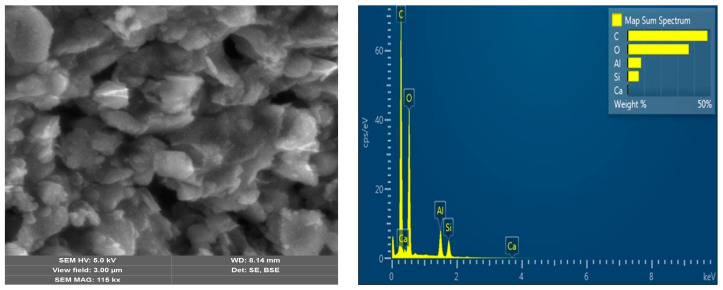
ZnIP microstructure after removal of Zn^2+^ ions before adsorption with elemental analysis.

**Figure 6:**
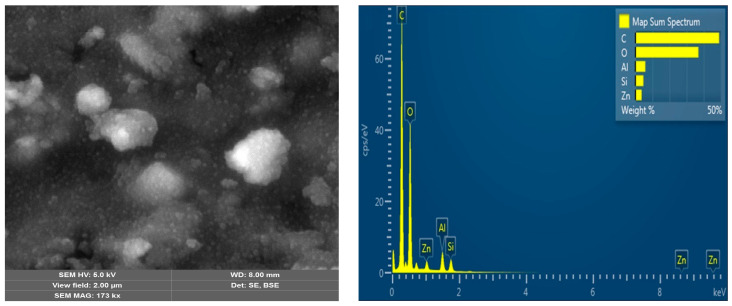
ZnIP microstructure after removal of Zn^2+^ ions after adsorption with elemental analysis.

**Figure 7:**
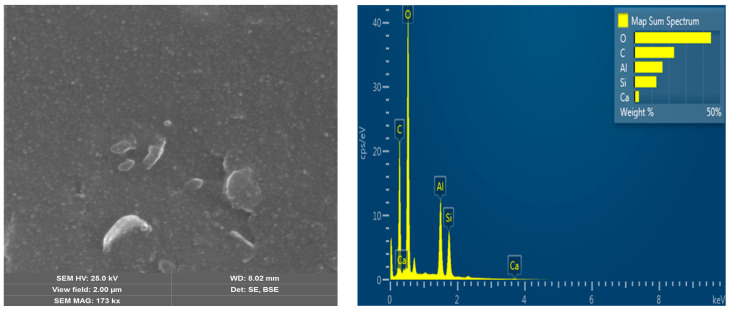
NIP microstructure with elemental analysis.

The surface morphology of ZnIP after the removal of Zn^2+^ ions from the polymer mesh before and after adsorption, as well as that of NIP, was studied using a scanning electron microscope (SEM) (Figure 5, Figure 6 and Figure 7). This study was conducted to analyze the changes in the ZnIP surface after the removal of Zn^2+^ ions from the polymer mesh and to evaluate its composition.

Microscopic images of the surface of the ZnIP sample after removal of Zn^2+^ ions from the polymer mesh before adsorption (Figure 5) show the presence of surface voids (prints). The removal of Zn^2+^ ions from the polymer network in ZnIP leads to the release of active polymer sites, creating optimal conditions for their capture from aqueous solutions during the adsorption process. The presence of such structural imprints is clearly visible in the micrographs (Figure 5). An analysis of the elemental composition and a multilayer EDS map confirm the absence of Zn^2+^ ions in microscopic images after their removal.

Micrographs of the surface of the ZnIP sample after removal of Zn^2+^ ions from the polymer mesh after adsorption (Figure 6) show that Zn^2+^ ions are successfully captured in the structural imprints of the polymer mesh, which eventually leads to their filling and fixation. This indicates the effective binding of Zn^2+^ ions to polymer imprints. The results of the analysis of the elemental composition and the multilayer EDS map confirm the appearance of Zn peaks on the ZnIP X-ray after adsorption.

On the micrographs of the surface of the NIP sample (Figure 7), a smoother and more heterogeneous surface is observed, without prints and structural changes characteristic of ZnIP. An analysis of the elemental composition and a multilayer EDS map confirm the composition of the NIP.

**Figure 8:**
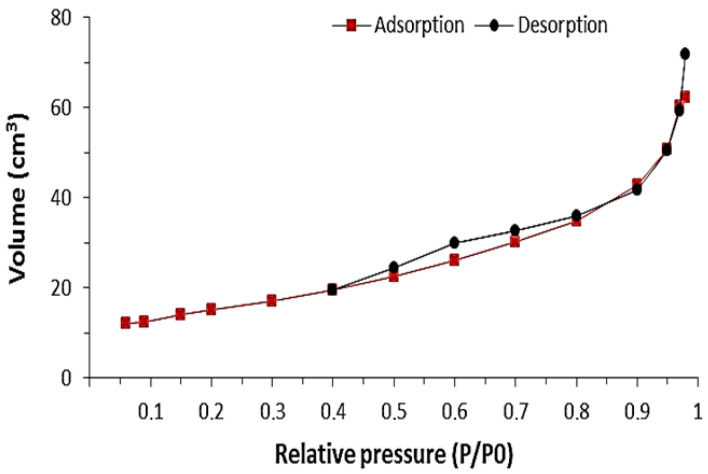
The adsorption/desorption isotherm of ZnIP.

**Figure 9:**
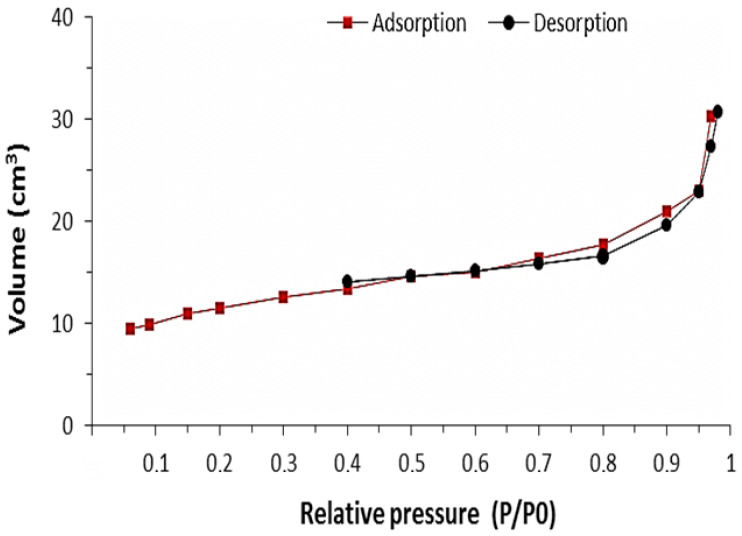
The adsorption/desorption isotherm of NIP.

Figure 8 and Figure 9 show the adsorption and desorption isotherms of ZnIP and NIP, determined by the low-temperature adsorption method.

**Figure 10:**
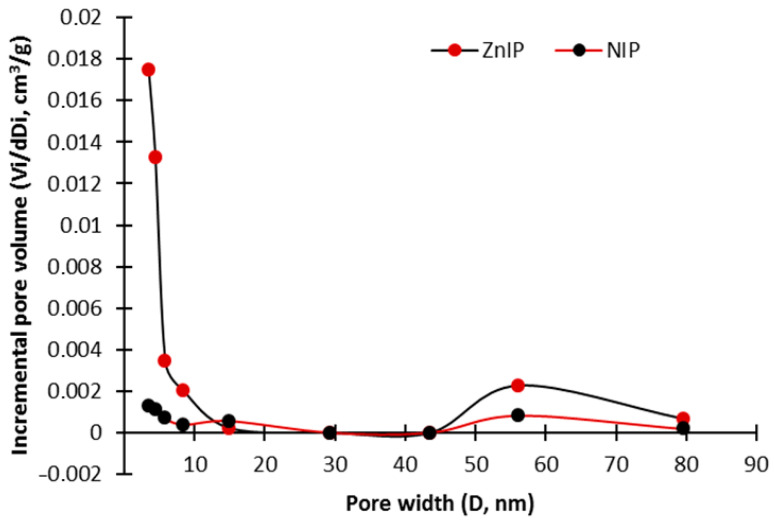
The distribution of ZnIP and NIP pore sizes.

Based on the analysis of the graphs of the adsorption/desorption isotherm ZnIP (Figure 8) and NIP (Figure 9), it can be concluded that their hysteresis loop corresponds to Type III, which is characteristic of materials with meso- and macropores with strong and weak adsorbate–adsorbent interactions, respectively. The ZnIP and NIP pore size distribution curves are shown in Figure 10.

Analysis of the distribution of ZnIP and NIP pore sizes has shown that imprinting has a significant effect on the structure of ZnIP, leading to significant changes in the distribution of pores. Imprinting leads to an increase in the total pore volume due to the uncorking of additional pores, which, in turn, significantly increases the adsorption capacity of ZnIP compared to NIP. According to the results of the analysis of the specific surface determined by the BET method, it was found that the average value of the specific surface of ZnIP was 40.60 ± 0.4 m^2^/g, while for NIP, it was 21.50 ± 0.3 m^2^/g.

### 3.2. Adsorption Study

**Figure 11:**
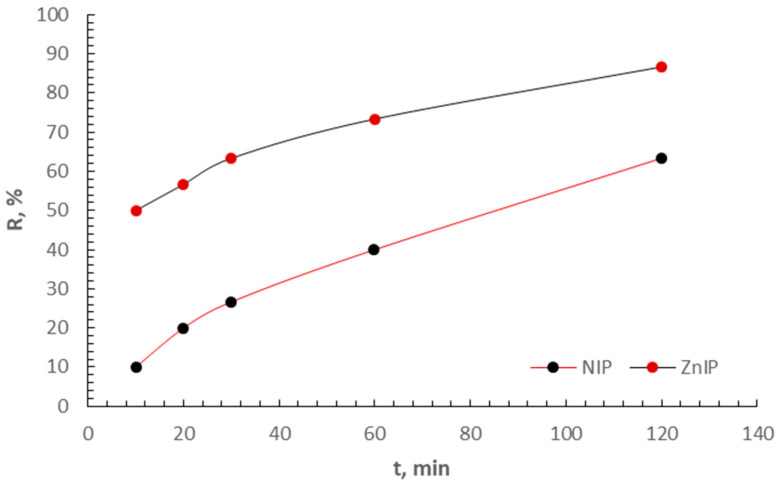
Kinetics of ZnIP and NIP sorption as a function of time.

Figure 11 shows the time dependences of the degree of sorption of Zn^2+^ ions by a zinc-imprinted polymer and a non-imprinted polymer.

Based on experimental data, the parameters of three types of linear dependencies were calculated, and correlation the coefficients were determined (Table 3).

The correlation coefficients obtained for the pseudo-first-order model demonstrate high accuracy in describing experimental data for both ZnIP (r = 0.9973) and NIP (r = 0.9970). This indicates that the kinetics of adsorption of Zn^2+^ ions on these polymers obeys the laws of the pseudo-first order. The value of the velocity constant for ZnIP exceeds that for NIP in all models, which is expressed in higher values of kinetic constants. This indicates the structural advantages of ZnIP, such as a large specific surface area, as well as optimized and better pore accessibility. Second- and third-order models can also be used to describe kinetics, but they exhibit lower accuracy, which limits their applicability in describing experimental data. The results obtained confirm the high efficiency of ZnIP in the processes of adsorption of Zn^2+^ ions in comparison with NIP.

**Figure 12:**
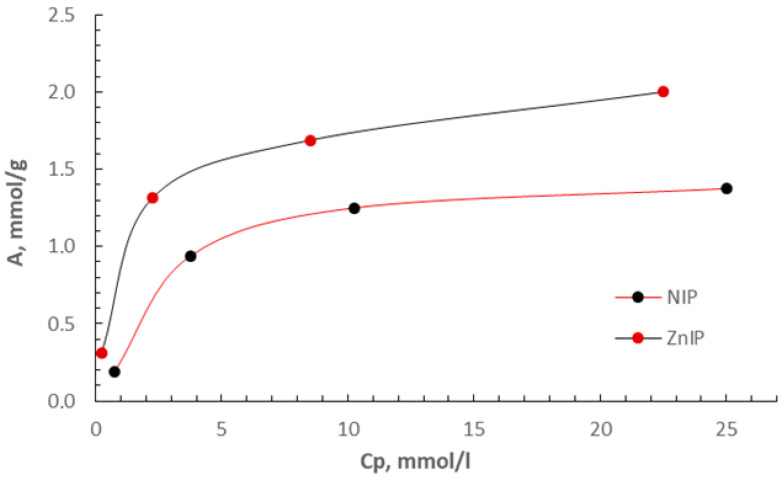
Isotherms of sorption of Zn^2+^ ions by ZnIP and NIP.

As is known, the quantitative characteristic of sorption is determined through Gibbs specific excess sorption, which can be calculated based on experimental data using Equation (1). To study the sorption capacity of ZnIP and NIP, we obtained isotherms of sorption of Zn^2+^ ions at a temperature of 298.0 K. The experimental data allowed us to evaluate the differences in sorption capacity and analyze the effect of the molecular structure of ZnIP and NIP on the adsorption process. The results are shown in Figure 12.

According to the results of the ZnIP and NIP sorption isotherms shown in Figure 12, ZnIP sorption isotherms demonstrate significant advantages in the processes of sorption of Zn^2+^ ions. The data show that the sorption capacity of ZnIP is significantly higher than that of NIP at all studied concentrations. This indicates the effectiveness of ZnIP in creating active centers for their interaction with Zn^2+^ ions.

The features of the ZnIP structure are related to the imprinting process. The removal of Zn^2+^ ions from the polymer mesh at the synthesis stage creates specific imprints. These prints play a key role, providing high selectivity and sorption capacity. During the sorption process, these prints are filled back with Zn^2+^ ions, which significantly improves the sorption efficiency. The results emphasize that ZnIP not only demonstrates a high sorption capacity, but also provides selectivity due to its specific interaction with Zn^2+^ ions.

When analyzing the sorption properties of ZnIP and NIP, not only the percentage of extraction of Zn^2+^ ions (R, %) was considered, but also the distribution coefficients (D) and the imprinting factor (IF), which were calculated in accordance with Equations (2)–(4). These parameters allow a deeper assessment of the sorption capacity and selectivity of ZnIP compared to NIP. The calculation results are shown in Table 4.

Based on the data in Table 4, it can be seen that ZnIP demonstrates significantly higher values of the distribution coefficient (D), which indicates its advantage in capturing Zn^2+^ ions from solution. In addition, the value of the imprinting factor (IF), calculated as the ratio of the distribution coefficients of ZnIP and NIP, indicates a high level of specificity of ZnIP to Zn^2+^ ions. These results confirm that the introduction of imprints into the ZnIP structure significantly increases the efficiency and selectivity of the sorption process, which makes this material promising for use in water purification tasks of heavy metal ions.

**Figure 13:**
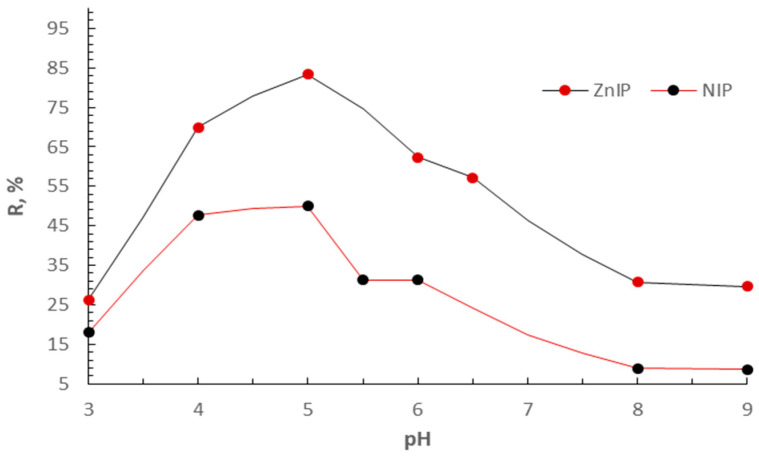
Dependence of the degree of extraction of Zn^2+^ ions by ZnIP and NIP on pH.

The adsorption of Zn^2+^ ions from aqueous solutions was studied in the pH range of 3.0–9.0 (Figure 13).

The nature of the pH dependence demonstrates that Zn^2+^ ions are successfully extracted by both ZnIP and NIP. However, the degree of extraction significantly depends on the pH of the solution. It follows from Figure 13 that the maximum degree of extraction of Zn^2+^ ions is observed in an acidic medium (pH < 5.0). With an increase in pH, a decrease in sorption capacity is observed, which is probably due to the hydrolysis of Zn^2+^ ions and the formation of zinc hydroxo complexes that are less susceptible to adsorption. These data confirm that the effective operation of ZnIP is possible in more acidic conditions, which must be taken into account when developing technologies for their application to purify aquatic environments. At the same time, the superiority of ZnIP over NIP is maintained throughout the studied pH range, which indicates the high selectivity of ZnIP to Zn^2+^ ions.

**Figure 14:**
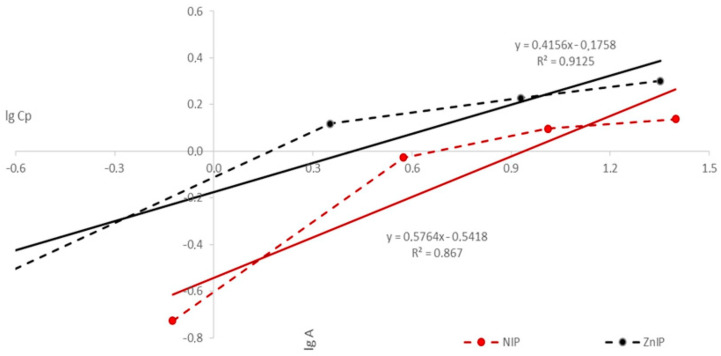
The isotherm of the sorption of Zn^2+^ ions on ZnIP and NIP in the coordinates of the linear form of the Freundlich equation.

Figure 14 shows the experimental data in the coordinates of the linear form of the Freundlich equation.

Based on the determination of linear regression coefficients, the parameters of the Freundlich equations for ZnIP and NIP were calculated (Table 5).

Equations (12) and (13) represent the processing of an array of experimental points according to the linear regression equation for Zn^2+^ ions by ZnIP and NIP, respectively:

(12) $$lgA=lg1.1832+\frac{1}{2.4060}\cdot {lgC}_{p}$$

(13) $$lgA=lg2.0526+\frac{1}{1.7348}\cdot {lgC}_{p}$$

**Figure 15:**
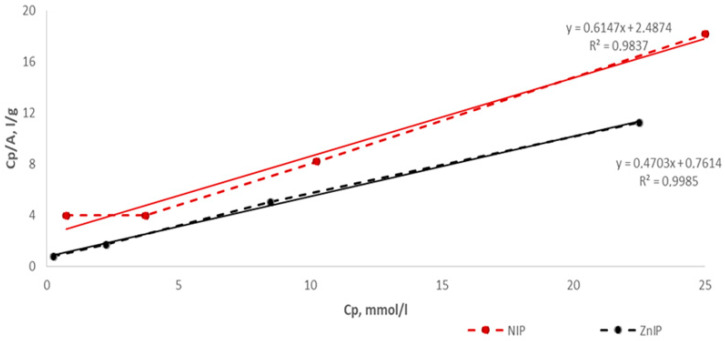
The isotherm of the sorption of Zn^2+^ ions on ZnIP and NIP in the coordinates of the linear form of the Langmuir equation.

Figure 15 shows the experimental data in the coordinates of the linear form of the Langmuir monomolecular adsorption equation.

Based on the determination of linear regression coefficients, the parameters of the equations were calculated—ion sorption constants and their maximum sorption per unit mass of ZnIP and NIP (Table 6).

Equations (14) and (15) represent the processing of an array of experimental points according to the linear regression equation for Zn^2+^ ions by ZnIP and NIP, respectively:

(14) $$\frac{C_{p}}{A}=\frac{1}{0.6177\cdot 2.1262}+\frac{1}{2.1262}\cdot C_{p}$$

(15) $$\frac{C_{p}}{A}=\frac{1}{0.2471\cdot 1.6269}+\frac{1}{1.6269}\cdot C_{p}$$

The high correlation coefficients for Equations (14) and (15) allow us to assert that the model Langmuir monomolecular adsorption equation adequately describes the isotherms of sorption of Zn^2+^ ions in the entire range of concentrations studied. The ZnIP shows a higher adsorption limit of 2.1262 mmol/g compared to NIP—1.6269 mmol/g. The adsorption equilibrium constant for ZnIP (0.6177 L/mmol) is also significantly higher than the value for NIP (0.2471 L/mmol). The correlation coefficients for the Langmuir model are r = 0.9985 for ZnIP and r = 0.9837 for NIP, which indicates an almost perfect fit of the data for ZnIP and a good match for NIP. These results confirm that adsorption most likely occurs on a homogeneous surface with the formation of a monolayer.

Freundlich’s model, on the contrary, demonstrates lower accuracy. The Freundlich constant (K_Fr_ = 1.1832) for ZnIP shows a slightly lower adsorbent capacity compared to NIP (K_Fr_ = 2.0526). The correlation coefficient is also lower: r = 0.9125 for ZnIP and r = 0.8670 for NIP, which indicates a less accurate correspondence of the data to the assumptions of this model.

The difference between the Langmuir and Freundlich models can be explained as follows. The Langmuir model assumes adsorption on a homogeneous surface with a fixed number of active centers and the formation of an adsorbate monolayer. High correlation coefficients confirm that these assumptions correspond to the adsorption process for both ZnIP and NIP. This means that the adsorption on the studied samples is limited to a single layer and the surface of the adsorbent is relatively homogeneous. The Freundlich model takes into account the possibility of adsorption on heterogeneous surfaces and the formation of a multilayer structure. However, lower values of correlation coefficients show that such processes are not dominant for these systems. This confirms that multilayer adsorption or strong heterogeneity of the surface does not play a significant role in this case.

Thus, ZnIP demonstrates better adsorption capacity and better compliance with the Langmuir model, which is confirmed by high correlation coefficients. These results indicate that adsorption on ZnIP occurs predominantly in a single layer and on a homogeneous surface. In addition, the high adsorption equilibrium constant and marginal adsorption for ZnIP emphasize its effectiveness at low concentrations of the adsorbate. NIP is also well described by the Langmuir model, but its adsorption capacity is lower than that of ZnIP. The Freundlich model describes adsorption on both samples less accurately, which is due to the predominance of monolayer adsorption on a homogeneous surface.

**Figure 16:**
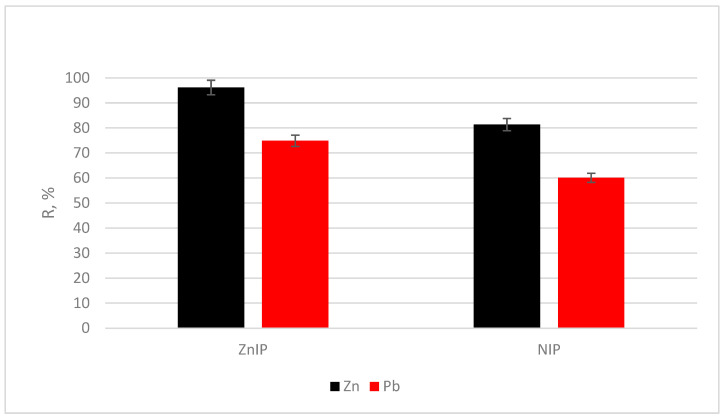
The degree of extraction of Zn^2+^ and Pb^2+^ ions by ZnIP and NIP.

To assess the selectivity of ZnIP and NIP, sorption was performed from a solution containing bimetal ions (Zn^2+^ and Pb^2+^), where Pb^2+^ ions were used as competitors with respect to Zn^2+^ ions. The experiments were carried out in static mode at a temperature of 22 ± 2 °C and pH = 5, which corresponds to the optimal conditions of the sorption process. Figure 16 shows the dependences of the degree of extraction (R, %) of Pb^2+^ and Zn^2+^ ions by ZnIP and NIP.

The results of this study (Figure 16) show that ZnIP and NIP have different adsorption efficiency for Zn^2+^ and Pb^2+^ ions. The degree of extraction of Zn^2+^ ions by ZnIP was 96.15%, which is significantly higher than the degree of extraction of Pb^2+^ ions (74.88%). The high selectivity of ZnIP for Zn^2+^ ions confirms the presence of active centers created during the imprinting process.

The degree of extraction of Zn^2+^ ions by NIP was 81.33%, which is lower than the same indicator for ZnIP. For Pb^2+^ ions, the recovery rate was 60.11%, which is also lower than that for ZnIP. The low selectivity indicators indicate the nonspecific nature of sorption on NIP.

A comparison of the data shows that ZnIP will prevail not only with a higher sorption capacity, but also with better selectivity with respect to Zn^2+^ ions. This is due to the presence of imprints that provide preferential binding of Zn^2+^ ions and minimize the sorption of Pb^2+^ ions. In the case of NIP, the adsorption of Zn^2+^ and Pb^2+^ ions occurs due to non-specific interactions, which limits its effectiveness in selective sorption tasks.

Table 7 shows the calculated values of the distribution coefficient (D), imprinting factor, selectivity coefficient (k) and relative selectivity coefficient (k’) for ZnIP and NIP.

The results of this study (Table 7) show that for both polymers, the values of the distribution coefficients for Zn^2+^ ions are higher than those for Pb^2+^ ions. This indicates the preferential interaction of both ZnIP and NIP with Zn^2+^ ions. The values of the imprinting factor for ZnIP significantly exceed those for NIP, which confirms the effectiveness of imprinting in creating specific active centers. ZnIP demonstrates a higher selectivity coefficient, which emphasizes its ability to bind Zn^2+^ ions in competition with Pb^2+^ ions. The relative selectivity coefficient of ZnIP is 2.90 times higher than that of NIP, which confirms its superiority in the selective sorption of Zn^2+^ ions.

The results obtained demonstrate that ZnIP has high efficiency and selectivity in the processes of sorption of Zn^2+^ ions from binary solutions containing competing Pb^2+^ ions. This is due to the specific imprints formed during the imprinting process.

To assess the possibility of reuse of synthesized ZnIP and NIP after adsorption from a binary solution containing Zn^2+^ and Pb^2+^ ions, experiments on their regeneration were carried out. The regeneration procedure was carried out by the acid hydrolysis method described in the experimental part. ZnIP and NIP regeneration was performed after each of the two adsorption cycles. After regeneration, the equilibrium concentrations of Zn^2+^ and Pb^2+^ ions in the filtrate were determined using an atomic emission spectrometer. The amount of adsorbed ions was calculated as the difference in concentration before and after sorption, related to the unit mass of the polymer.

The results of this study show that after the first cycle, the adsorption capacity of ZnIP and NIP remained at a high level. After the second cycle, the adsorption capacity of ZnIP decreased to 40%, and that of NIP to 20% of the initial value. Based on the studies conducted, it was found that the adsorption capacity of ZnIP and NIP significantly decreases after two regeneration cycles. This may be due to the partial degradation of the prints or to a change in the polymer structure during acid hydrolysis. In this regard, it is advisable to limit the number of regeneration cycles to two in order to maintain an acceptable efficiency of adsorbents.

## 4. Conclusions

Thus, during this work, ZnIP and NIP were synthesized using the molecular imprinting method. The novelty of the work lies in the use of humic acids isolated from coals of the Shubarkol deposit (Karaganda, Kazakhstan) as a basis for the imprinted polymer matrix, methacrylic acid and ethylene glycol dimethacrylate as a functional monomer and crosslinking agent, respectively. The composition and structure of the synthesized ZnIP and NIP were confirmed using modern methods of physicochemical analysis. The results of the DTA showed that both polymers have high stability in thermal processes. Studies of the specific surface area and pore volume conducted using the Sorbi-MS adsorption analyzer revealed a significant effect of imprinting on the ZnIP structure. The analysis of the pore size distribution showed that the imprinting process leads to changes in the porous structure of ZnIP, which contributes to the creation of specific imprints for the sorption of Zn^2+^ ions. During the study of the sorption properties of ZnIP and NIP, it was found that ZnIP exhibits a higher sorption capacity with respect to Zn^2+^ ions compared to NIP. The maximum adsorption of Zn^2+^ ions was 2.1262 mmol/g for ZnIP, which is significantly higher than that for NIP (1.6269 mmol/g). Kinetic studies have shown that the sorption of Zn^2+^ ions on both ZnIP and NIP is described by a pseudo-first order equation, which confirms the correspondence of the model. Under the conditions of sorption from a binary solution (Zn^2+^ and Pb^2+^), ZnIP demonstrated better selectivity and higher sorption capacity compared to NIP. The relative selectivity coefficient of ZnIP turned out to be 2.90 times higher than that of NIP. Regeneration experiments have shown that after two cycles, the adsorption capacity of ZnIP decreases to 40%, and that of NIP to 20%. This indicates the expediency of using no more than two regeneration cycles in order to maintain their effectiveness.

The results obtained confirm that ZnIP is a promising sorption material for the selective removal of Zn^2+^ ions from complex multicomponent systems such as wastewater or industrial solutions. High selectivity, efficiency and stability make ZnIP a valuable tool for solving environmental and technological problems related to the removal of heavy metals.

## Figures and Tables

**Table 1:** Initial components for the synthesis of zinc-imprinted and non-imprinted polymers.

Polymer	HA, (mmol)	MAA, (mmol)	Zn(CH_3_COO)_2_, (mmol)	EGDMA, (mmol)	BP, (mmol)
ZnIP	1	1	1.00	10	0.1
NIP	1	1	–	10	0.1

**Table 2:** Characteristics of zinc-imprinted and non-imprinted polymers.

Polymer	C^g^, %	H^g^, %	N^g^, %	O^g^, %	Σ(COOH+OH) mg-eq/g	Yield, %
ZnIP	61.32 ± 0.2	3.97 ± 0.1	0.67 ± 0.1	34.04 ± 0.3	4.43 ± 0.2	76.54
NIP	57.32 ± 0.2	3.85 ± 0.1	0.67 ± 0.1	38.16 ± 0.3	4.95 ± 0.2	77.98

**Table 3:** Kinetic equations of sorption of Zn^2+^ ions ZnIP and NIP at T = 298.0 K and pH = 5.0 and correlation coefficients (r).

The Pseudo-Order of the Reaction	Sorbed Ion Zn^2+^ Zinc-Imprinted Polymer		
First	lnC = 1.4083 − 0.0118t	r = 0.9973	k_1_ = 0.0118
Second	1/C = 0.1665 + 0.0067t	r = 0.9813	k_2_ = 0.0067
Third	1/C^2^ = 0.1001 + 0.0085t	r = 0.9341	k_3_ = 0.0085
**The Pseudo-Order of the Reaction**	**Sorbed Ion Zn^2+^ by a Non-Imprinted Polymer**		
First	lnC = 1.9653 − 0.0079t	r = 0.9970	k_1_ = 0.0079
Second	1/C = 0.1234 + 0.0019t	r = 0.9863	k_2_ = 0.0019
Third	1/C^2^ = 0.0047 + 0.001t	r = 0.9571	k_3_ = 0.0010

**Table 4:** Extraction rate (R, %), distribution coefficient (D) and imprinting factor (IF).

R, %		D·10^−2^		IF
ZnIP	NIP	ZnIP	NIP	
83.33	50.00	12.50	2.50	5.00
70.00	50.00	5.83	2.50	2.33
44.26	32.79	1.99	1.22	1.63
26.23	18.03	0.89	0.55	1.62

**Table 5:** Linear approximation coefficients (y = kx + b), Freundlich equation parameters and correlation coefficients (r).

Polymer	k	b	K_Fr_	n	r
ZnIP	0.4156	−0.1758	1.1832	2.4060	0.9125
NIP	0.5764	−0.5418	2.0526	1.7348	0.8670

**Table 6:** Linear approximation coefficients (y = kx + b), Langmuir equation parameters and correlation coefficients (r).

Polymer	k	b	Maximum Specific Adsorption, A_∞_, mmol/g	Adsorption Equilibrium Constant, K_L_, L/mmol	r
ZnIP	0.4703	0.7614	2.1262	0.6177	0.9985
NIP	0.6147	2.4874	1.6269	0.2471	0.9837

**Table 7:** The values of the distribution coefficient, imprinting factor, selectivity coefficient and relative selectivity coefficient for ZnIP and NIP.

Ions	D·10^−2^		IF		k		k’
	ZnIP	NIP	ZnIP	NIP	ZnIP	NIP	
Zn^2+^	2.50	0.44	5.73	–	–	–	–
Pb^2+^	0.30	0.15	1.98	–	8.38	2.89	2.90

## Data Availability

The original contributions presented in this study are included in the article. Further inquiries can be directed to the corresponding author.
